# Management and Outcomes of Non-Missile Penetrating Brain Injury Involving the Anterior Skull Base: A Case Report and Systematic Review

**DOI:** 10.3390/jcm14165731

**Published:** 2025-08-13

**Authors:** Wojciech Czyżewski, Michał Szymoniuk, Jakub Litak, Krzysztof Kura, Klaudia Kuś-Budzyńska, Aleksandra Dryla, Jacek Baj, Kamil Torres, Grzegorz Staśkiewicz

**Affiliations:** 1 Department of Neurosurgery, Maria Sklodowska-Curie National Research Institute of Oncology, ul. W.K. Roentgena 5, 02-781 Warsaw, Poland; wojciech.w.czyzewski@gmail.com; 2 Department of Didactics and Medical Simulation, Medical University of Lublin, Chodźki 4, 20-093 Lublin, Poland; kamiltorres@wp.pl; 3 Department of Neurosurgery, Medical University of Lublin, Jaczewskiego 8, 20-090 Lublin, Poland; krzysztof_kura@wp.pl (K.K.); kkusbudzynska@gmail.com (K.K.-B.); drylaaleksandra@gmail.com (A.D.); 4 Department of Clinical Immunology, Medical University of Lublin, Chodźki 4a, 20-093 Lublin, Poland; jakub.litak@gmail.com; 5 Department of Correct, Clinical and Imaging Anatomy, Medical University of Lublin, Jaczewskiego 4, 20-090 Lublin, Poland; jacek.baj@umlub.edu.pl; 6 Department of Plastic, Reconstructive Surgery with Microsurgery, Medical University of Lublin, Jaczewskiego 8, 20-090 Lublin, Poland; 7 1st Department of Radiology, Medical University of Lublin, Jaczewskiego 8, 20-090 Lublin, Poland; grzegorz.staskiewicz@gmail.com

**Keywords:** penetrating head injury, crossbow, traumatic brain injury, case report, systematic review

## Abstract

**Introduction:** Non-missile penetrating brain injury (PBI) involving the anterior skull base constitutes a rare subclass of traumatic brain injury in civilians. Management of this type of trauma is poorly described in the literature, with only case series and reports available. **Materials and Methods:** A systematic search was conducted across PubMed, Scopus, and Web of Science databases. The study included reports of adult patients with non-missile PBI with foreign bodies crossing the anterior skull base, published between the years 2000 and 2024. The patients were divided into three groups based on the entry point of foreign bodies: transorbital, transmental, and transnasal injuries. The obtained data were analyzed through descriptive statistics. A case report of a 20-year-old male following PBI involving the anterior skull base caused by suicidal self-shooting with a crossbow is presented. **Results:** A total of 17 articles reporting 40 patients and the current case were included. The mean age of the patients was 37.4 ± 13.1 years, and 92.7% of them were male. Transorbital injury was the dominant type of PBI (29 cases), followed by transmental injury (7 cases) and transnasal injury (5 cases). A total of 37 patients (90.2%) were managed operatively due to retained foreign bodies after PBI. Antibiotic prophylaxis was implemented in 33 cases (80.5%), mostly in transorbital (93.1%) and transnasal (100%) PBI. In seven reported cases, antiepileptic drugs were preventively administered. At the last follow-up, 18 patients (47.4%) did not fully recover neurological functions, with vision loss as the most common deficit. **Conclusions:** Management of non-missile PBIs involving the anterior part of the skull base is complex, challenging, and often requires a multidisciplinary team including neurosurgeons, ENT surgeons, ophthalmologists, and maxillofacial surgeons. In this type of traumatic brain injury, following proper management may lead to favorable outcomes with minimal neurological deficits.

## 1. Introduction

Among traumatic brain injuries in the civilian population, penetrating brain injury (PBI) represents a relatively rare type of injury, constituting about 10% of them [1]. PBIs can be classified into two main categories according to the velocity of the penetrating object: high-velocity injuries (often caused by missiles or bullets) and low-velocity (non-missile) injuries [2]. In peacetime conditions, non-missile PBIs are more common and are usually caused by pointed or sharp objects such as metal rods, wooden sticks, knives, screwdrivers, and bow or crossbow projectiles [3]. Although non-missile PBIs are characterized by better outcomes compared with missile PBIs due to less diffuse primary injury and lack of cavitation, they remain a life-threatening entity [4,5]. Moreover, non-missile PBIs often coexist with severe orbital, craniofacial, or skull base injuries, making their management more complex and challenging. They can also cause fatal complications such as infection of the central nervous system (CNS), cerebrospinal fluid (CSF) leakage, pneumocephalus, or injuries of major vascular structures [6].

Skull base fractures can be classified into three types based on anatomical location: anterior, middle, and posterior [7]. Injuries of the anterior part of the skull base are characterized by distinct clinical features, management requiring a multidisciplinary team, and different outcomes compared with injuries of the middle and posterior parts [8]. A non-missile PBI penetrating through the anterior skull base constitutes an uncommon entity, and hence, its management and outcomes are scarcely described in the available literature. Furthermore, to the best of our knowledge, no systematic review has been published on this topic. Therefore, available case series and reports of non-missile PBI involving the anterior skull base are summarized in the current paper. Moreover, we would like to present a case of a suicidal patient treated in our department due to a PBI caused by self-shooting with a crossbow. This case demonstrates a novel use of a solution of sodium hypochlorite and hypochlorous acid (SHC/HCA-G) solution for intraoperative irrigation as an antiseptic agent.

## 2. Case Description

### 2.1. Clinical Presentation

**Figure 1:**
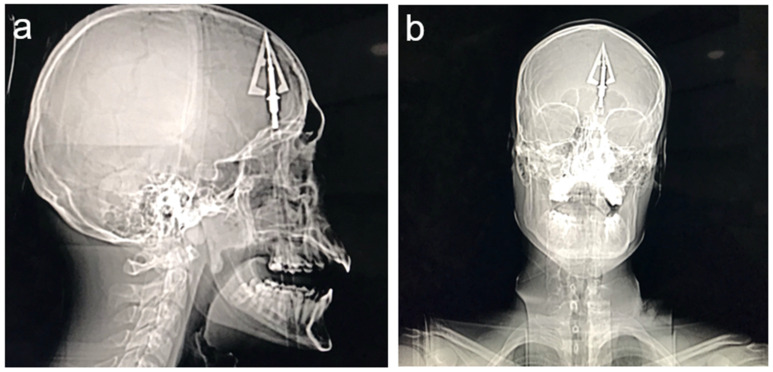
Computed tomography scan images showing the retained crossbow projectile: (**a**) lateral view and (**b**) anterior–posterior view.

A 20-year-old male patient was brought to the emergency department after self-shooting with a hunting bow, most probably as a part of a suicide attempt. Computed tomography (CT) displayed an arrow-shaped foreign body that pierced the fundus of the oral cavity, palate, nasal cavity, left ethmoid bone, left frontal lobe, and halted at the cranial calvaria slightly lateral to the superior sagittal sinus (Figure 1). Neurological examination did not reveal any evident abnormalities, as the patient could logically communicate with medical personnel. Regarding cranial nerve evaluation, only olfactory function seemed to be impaired. Muscle strength was preserved in all extremities. Sensation to light touch and proprioception was intact. Deep tendon reflexes were symmetric and without pathological signs.

Immediately, he was transferred to the neurosurgical operating room and operated on by a hybrid neurosurgical–ENT team.

### 2.2. Surgical Procedure

**Figure 2:**
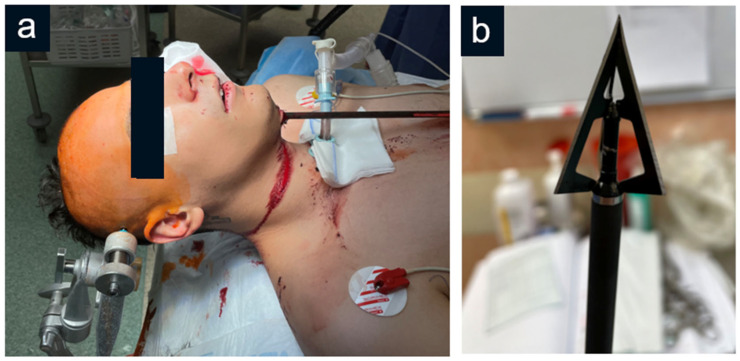
(**a**) Preoperative photograph of the patient with a retained crossbow projectile passing through the submental area; (**b**) the tip of the crossbow projectile retrieved from the patient’s head.

First, a tracheostomy was performed by an ENT surgeon. Then, after careful preparation of the operating field and skin decontamination with a solution of SHC/HCA-G at concentrations of 50 ppm/50 ppm for NaOCl and HOCl (Granudacyn^®^), decompressive bifrontal craniectomy was performed. Afterward, the arrow point was detached from the shaft by unscrewing it and then it was safely removed from the left frontal lobe. Subsequently, anterior fossa autoplasty was performed with the use of hemostatic materials, i.e., TachoSil^®^. Because of the unsterile environment and extremely high risk of postoperative infection complications, again, Granudacyn^®^ was used copiously for operative field irrigation (in a total volume of 500 mL). The bone flap was not restored, and the skin was closed in a routine fashion (Figure 2).

### 2.3. Postoperative Course

After surgery, the patient was transferred to the ICU, where he received infection prophylaxis with wide-spectrum antibiotics intravenously (2 g ceftriaxone with 1 g vancomycin; twice per 24 h). Simultaneously, his wounds were cleaned with Granudacyn^®^ applied topically each day and healed satisfactorily. Then, he was moved to the Department of Rehabilitation, where the patient was decannulated and verticalized. Postoperative examination revealed olfactory dysfunction and no other neurological deficits.

Due to an extensive coughing reflex during the postoperative period, the patient subsequently developed pneumocephalus caused by gas escape from the nasal cavity through ethmoid fractures to the anterior cranial cavity and qualified for reoperation. De novo tracheostomy was performed, followed by anterior cranial fossa reconstruction with pericranium and titanium meshes. Again, intraoperative cleansing with sodium chloride-hypochlorous acid solution was implemented. Swab results taken intraoperatively revealed no brain tissue contamination.

Following the secondary procedure, the patient recovered rapidly and, apart from a mild body temperature increase, did not present any infectious complications. After two months of hospitalization prolonged by a coronavirus infection, he was discharged from the department in a neurologically intact state. At the 6-month follow-up, the patient still demonstrated no sensory and motor deficits.

## 3. Systematic Literature Review

### 3.1. Materials and Methods

The literature review was conducted following the Preferred Reporting Items for Systematic Reviews and Meta-Analyses (PRISMA) 2020 guidelines (see Supplementary File S1 for the PRISMA checklist). The review was not registered, and the review protocol was not prepared. A systematic search was conducted across PubMed, Scopus, and Web of Science databases on 15 October 2024 with the use of a search strategy developed by the authors for PubMed database search (Table 1) and adapted into the remaining databases by the Polyglot Search Tool (https://sr-accelerator.com/#/polyglot (accessed on 14 October 2024)). Relevant studies retrieved using the search strategy were imported into Mendeley Reference Manager for reference management and Rayyan software for study screening and selection. Moreover, reference lists of the included articles were hand-searched to identify additional studies.

Records were included if they reported patients after non-missile PBI with the trajectory of a foreign body crossing through the anterior part of the cranial base and published between the years 2000 and 2024. Eligible study designs were as follows: prospective and retrospective cohort studies, case–control studies, pre–post treatment studies, retrospective studies, case series, and case reports. Studies were excluded if they were post-mortem analyses, reviews, meta-analyses, commentaries, editorials, animal studies, preclinical studies, written in a language other than English, or reported patients aged below 18.

Screening of the titles and abstracts and full-text screening were performed independently by two authors. Discrepancies between the assessments of both authors were discussed with the first author. The articles were screened according to the above eligibility criteria.

The age and gender of the patient, clinical presentation, mechanism of injury, cause of injury, trajectory of foreign body, type of imaging and treatment method applied, neuroinfection prophylaxis used, complications, and outcome at the last follow-up were extracted from each included article. Two authors independently conducted the data extraction process. Accuracy of data extraction was verified by the first author, and any discrepancies were resolved through discussion with other authors. The patients reported by eligible articles were additionally divided into three groups according to the entry point of the penetrating foreign body: transorbital injuries for the orbital area, transmental injuries for the submental area, and transnasal injuries for the nasal cavity. Risk of bias of included studies was assessed using the JBI’s critical appraisal checklist tool for case reports. The obtained data were analyzed through descriptive statistics. Continuous variables were presented with means and standard deviations, whereas dichotomous variables were summarized with the use of frequencies and percentages. Statistical analysis was performed using Statistica software (Statistica version 13.0, TIBCO Software Inc., Palo Alto, CA, USA).

### 3.2. Results

#### 3.2.1. Search Results

**Figure 3:**
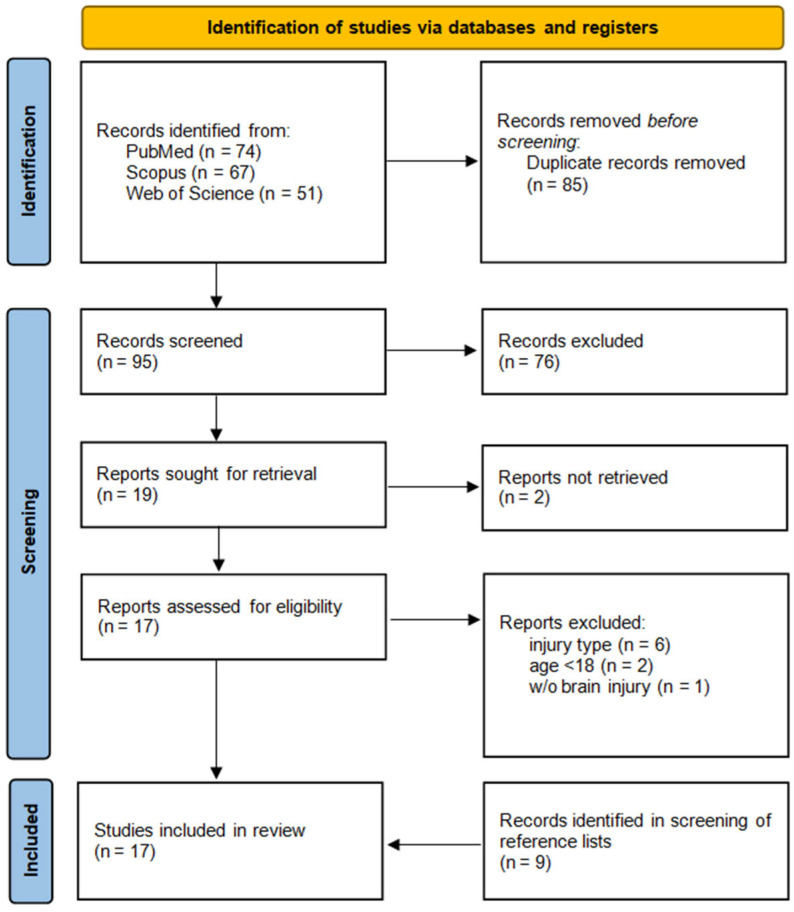
PRISMA flow diagram of study selection.

A total of 192 records were found, including 74 in PubMed, 67 in Scopus, and 51 in the Web of Science database. After removing duplicates, 95 records remained for screening. Selection based on titles and abstracts identified 19 relevant articles. A total of 17 full texts that were retrieved were further analyzed to see if the papers fulfilled the eligibility criteria. This consequently led to the exclusion of 9 studies due to the following causes: anterior part of skull base not injured (n = 6), age of patient below 18 (n = 2), and patient without traumatic brain injury (n = 1). Additional screening of reference lists identified 9 articles. Finally, 17 studies and current case reports matched the eligibility criteria, including a total of 41 patients (Figure 3).

#### 3.2.2. Characteristics of Included Articles

The articles included in the current systematic review were published between 2011 and 2024. Cases reported by the authors from China were the most common (21 patients, 51.2%), followed by Indonesia (5 patients, 12.2%), and the United States (4 patients, 9.8%). Among causes of injury, wooden sticks were the most common (8 patients, 19.5%). Other foreign bodies causing PBIs reported in eligible articles included crossbow projectiles, metal rods, knives, bamboo sticks, tree twigs, nails, rifle rods, sugarcane, garden poles, pressure cooker nozzles, electric drills, metal arrows, wooden stakes, and fishing accessories. Imaging modalities used included CT, plain radiographs, computed tomography angiography (CTA), magnetic resonance (MRI), and digital subtraction angiography (DSA). Surgical management was performed in 37 patients (90.2%) with a multidisciplinary team in some cases involving ENT surgeons, ophthalmologists, or maxillofacial surgeons (Table 2).

#### 3.2.3. Quality Assessment of Included Articles

Table 3 presents the results of the risk of bias assessment according to the JBI’s critical appraisal checklist tool for case reports. In terms of the description of demographic characteristics (domain 1), current clinical condition (domain 3), diagnostic methods (domain 4), and adverse events (domain 7), all articles fulfilled the required criteria. Patient’s history (domain 2) was clearly described in 12 of the 17 articles. Fourteen papers met the minimal requirements regarding the description of the surgical procedure (domain 5). In 14 articles, post-intervention follow-up (domain 6) was reported sufficiently. Finally, takeaway lessons (domain 8) were provided by ten articles.

#### 3.2.4. Characteristics of Patients with Non-Missile PBIs Involving the Anterior Skull Base

The mean age of the patients was 37.4 years (SD: 13.1), ranging between 18 and 75 years. Thirty-eight patients (92.7%) were male, and three remaining patients were female (7.3%). Transorbital injury was the dominant type of non-missile anterior skull base PBI (29 cases), followed by PBIs with an entry point at the submental area (7 cases) and PBIs entering through the nasal cavity (5 cases). A foreign body was retained in the injury site in 38 cases (92.7%). The most frequent mechanism of injury was an accident (28 patients, 68.3%), followed by violence (7 patients, 17.1%) and a suicide attempt (6 patients, 14.6%). The patients on admission were neurologically intact in most cases (30 patients, 73.2%), and scored 15 points on the Glasgow Coma Scale (GCS) (20 patients, 54.1%) (Table 3). Among reported neurological deficits, loss of vision and other dysfunctions of the optic apparatus were most commonly observed. Severely altered consciousness or major neurological symptoms such as hemiplegia were only observed in single cases (Table 4).

#### 3.2.5. Management and Outcome

CT was the most frequently used imaging modality (97.6% of patients), followed by MRI (43.9%) and CTA (12.2%) (Table 5).

A total of 37 patients (90.2%) were managed operatively, mostly by means of craniotomy and craniectomy in other patients, due to the retained foreign body after PBI. In three cases, the penetrating foreign body was not retained at the injury site, and all of those patients were managed non-operatively. In one patient, despite a foreign body present at the injury site, conservative treatment with broad-spectrum antibiotics was chosen with no infectious complications [9].

Prophylaxis of neuroinfection was implemented in 33 cases (80.5%), mostly in transorbital (93.1%) and transnasal (100%) PBIs, whereas only one patient with transmental PBI (14.3%) received antibacterial prophylaxis. Antibiotics utilized by authors of reported cases included cephalosporins, metronidazole, vancomycin, linezolid, biapenem, sulbactam, aminoglycosides, and piperacillin. Some authors additionally used amphotericin B as antifungal prophylaxis.

Irrigation of the operative field for infection prophylaxis purposes was performed in two cases—in one of them, the authors utilized 0.9% NaCl solution with broad-spectrum antibacterial agents, and in the remaining, the current case, a novel antiseptic solution containing SHC/HCA-G was used.

In seven reported cases, antiepileptic drugs (AEDs) for seizure prevention were utilized. Two patients received sodium valproate, and another two subjects received phenytoin. The authors of three reports did not specify the antiseizure drug used.

At the last follow-up, 20 patients were neurologically intact (52.6%), and 18 patients did not recover completely from injury (47.4%). Loss of vision was the most common persistent neurological deficit.

#### 3.2.6. Complications

The most commonly reported complication was CSF leak, which occurred in nine patients (22.0%). Four of them suffered from transmental PBI, another three patients suffered from transorbital PBI, and the remaining two patients presented with transnasal PBI. All cases of CSF leak were managed by repairing the skull base defect with an additional endoscopic endonasal approach.

Intracranial hemorrhage was reported in six patients (14.6%). Subarachnoid hemorrhage occurred in two patients [10]. Another two patients were diagnosed with intraparenchymal hemorrhage [11,12], and one patient presented with a subdural hemorrhage [12]. Moreover, one patient developed severe traumatic brain injury with combined subdural, intraparenchymal, and intraventricular hemorrhage [10].

Three patients (7.3%) developed pneumocephalus. These patients were reported by two authors [13,14] and the current case.

Vascular complications occurred in two patients (4.9%)—one patient was diagnosed with an internal carotid artery tear [15] and another one with traumatic cavernous fistula [12].

Infectious complications and epilepsy were rare in the reported cases—only one patient developed a brain abscess [9] and one patient was diagnosed with epilepsy [12].

## 4. Discussion

Traumatic brain injuries can be classified into closed and penetrating injuries, with the former being far more common. A PBI is often caused by sharp objects penetrating and damaging brain tissue through the skull bone and dura mater [16], usually affecting young and middle-aged males [6].

PBIs can be further divided into missile and non-missile injuries. The first of them causes damage due to high kinetic and thermal energy, while the latter injury mainly results in local destruction of brain tissue. Both are associated with secondary damage that mostly consists of vascular lesions as well as infections [6,17,18].

Stab wounds are the most common cause of non-missile PBI involving foreign bodies such as metal rods, knives, bamboo sticks, tree twigs, nails, rifle rods, garden poles, electric drills, wood stakes, and even sugarcane, pressure cooker nozzles, or fishing accessories, according to the results of the current study. However, the use of some weapons is occasionally documented in modern-era literature, and in the current systematic review, crossbow projectiles and metal arrows were also reported as penetrating foreign bodies. The crossbow, as in the case presented in this paper, is one of the less common causes of PBI, particularly in developed countries [19]. They are usually related to accidents, but are seldom used as part of suicide attempts, especially with mental illness occurrence [20]. Nevertheless, these injuries are still being reported in Asian or African countries, especially in tribal environments, as displays of violence [21,22]. Crossbow projectiles may reach relatively high velocities ranging from 45 to 67 m/s [23]. However, due to the high sharpness of the arrowhead, the primary damage is limited to dissected tissues and does not harm the large volume of brain tissue, as in the case of missiles or bullets.

On admission, most of the patients were neurologically intact and scored 15 points in GCS. Therefore, it is very important to perform a comprehensive examination of the patient with suspected PBI, as in some reported cases, retained foreign bodies were not visible at first glance [9,24], which can delay diagnosis and appropriate treatment.

In the non-missile anterior skull base, PBI foreign bodies mostly entered through the orbit (29 patients), and this was associated with the accidental character of injury. Moreover, the low thickness of orbital bone makes this region susceptible to penetration by foreign bodies into the anterior cranial fossa [25], contributing to a high percentage of these injuries in the current study. On the other hand, the transmental trajectory involves more structures, such as the palate and ethmoid bone, providing more protection. Moreover, due to the anatomical location floor of the oral cavity, it is rarely available for accidental penetrating injuries. This causes the need for higher velocities of foreign bodies and often intentional action. Our results reflect these findings, demonstrating only seven cases of transmental injuries, whereas six of them had suicidal character.

Following careful clinical examination, imaging investigations are necessary to establish a proper diagnosis. CT is the best modality for these injuries, as it better visualizes foreign body trajectory with 3D-reconstruction images compared with plain radiographs, can detect coexisting hematomas, and aids in preoperative planning [9]. However, the utility of CT scans is limited in cases of non-metallic foreign objects. In this regard, MRI scans can be useful. In case of suspected vascular injury, DSA or CTA is highly recommended [3,9].

In the presence of a foreign body retained at the injury site, surgical management should be implemented in the first 12 h following PBI, as available studies suggest [5,9]. The same period for surgical intervention was proposed in missile PBI management [26]. Prolonged retention of the object increases the risk of infectious complications and seizures [12]. Other indications for surgery include CSF leakage, vascular injuries, and intracranial hemorrhages [9]. Surgical management of non-missile PBI involving the anterior skull base constitutes a challenging matter because removal of the foreign body requires appropriate skills and is associated with a 10% risk of mortality [27]. Incorrect surgical maneuvers may damage vital brain regions and worsen the clinical outcome. Moreover, in some cases, the foreign body can subside deep inside the brain parenchyma over time, influenced by the gravitational force, causing more extensive injury [11]. Hence, this finding emphasizes the appropriate timing of surgical intervention.

Infectious complications may occur after the incidence of PBI, such as brain abscess, meningitis, encephalitis within the CNS, or outside the CNS, as wound or bone flap infections [26]. They are related to contact with foreign bodies outside the CNS origin, such as hair, skin, or bone fragments [5]. They are observed more often in cases presenting CSF leakage, trans-ventricular penetration, air sinus involvement, or injuries involving the contralateral hemispheres [28]. The timespan between the injury and infectious complication ranges mainly from 3 (55% of cases) to 6 weeks (90% of cases), although some documented cases present an unusual clinical presentation. In one case, a brain abscess was diagnosed after a 47-year latency period [29].

The rate of neuroinfection following PBIs differs between studies. In the multicenter study conducted in the years 2006–2016 and performed on 763 patients with PBI, 7% developed a postsurgical infection [30]. Although the contribution to higher mortality and morbidity is well documented, there is a lack of universal guidelines on their prevention.

The most common approach, typically chosen by clinicians to prevent infectious complications, involves the use of intravenous antibiotics, as well as intraoperative lavage and irrigation with antimicrobial agents (including antibiotics and antiseptics). Regardless of an overall consensus on this treatment, recent evidence-based medicine has focused on the preponderance of different agents is uncertain and requires further investigation [31].

In a review of the literature carried out by Kerrin Sunshine et al., presented at the Congress of Neurological Surgeons 2020, out of 903 patients from high-income countries, 64% received prophylaxis with oral or IV antibiotics. Among them, 8.32% developed an infection, which in juxtaposition comprises 6.75% of patients who did not receive the prophylactic agents [32]. Although antibiotics do not dramatically reduce the risk of infection according to the cited study, there are other factors, such as intracranial pressure monitors or surgical intervention, that contribute to the higher risk. Surprisingly, neither the degree of dural penetration nor the trajectory of the oropharynx altered the risk of neuroinfection [30]. A different approach was presented by Sayed Faraz Kazim et al. in “Guidelines for the Management of Penetrating Brain Injury”, citing a large multi-center study from 1991, where the use of broad-spectrum antibiotics helped lower infection risk to the level of 1–5% [5,33]. Although antibiotic application differs between centers, broad-spectrum antibiotics, including cephalosporines, penicillin, or metronidazole, generally are most extensively used [30,34].

The factor that additionally increases the rate of neuroinfection is the leakage of the CSF due to basal fractures and subsequent tear of the dura mater. According to Aarabi B. in a study regarding head injuries that occurred in the Iran–Iraq war, concerning mostly gunshot wounds, the rate of this complication reached around 8.7%, with 36% of them aggravated by the infection [35]. This, in turn, increases the 5-fold risk. Luckily, in the present case, there was no leakage of the CSF.

In the local department, the operating room routine involves perioperative focal use of an antiseptic agent—Skinsept Color, which consists of ethanol (96%), isopropyl alcohol, benzyl alcohol, and accessory substances. According to some studies, there is no significant difference in reducing rates of postoperative surgical site infection (SSI) between popular agents [36,37]. However, newer studies show differences between the efficacy of agents, which also change depending on the type of surgery performed [38]. Nevertheless, in the current case, it was decided to subsequently use SHC/HCA-G (Granudacyn^®^), precautiously on the surgical site, having in mind the significantly greater risk of postoperative infection in the patient due to contamination of the arrow as well as basal skull fractures.

Another way to prevent SSI is the use of antiseptic agents intraoperatively with irrigation of the surgical site. Since in the current literature, there is a lack of definitive studies presenting the advantages of available options, especially in brain surgeries, the proper choice of them remains challenging [39]. Among plenty of accessible options, local department workers usually use sodium chloride, hydrogen dioxide, as well as a solution of SHC/HCA-G (Granudacyn^®^) in contaminated wounds. According to Anna-Lena Severing et al., among SHA/HCA products, SHC/HCA-G (Granudacyn^®^) solution’s concentration has preferable biocompatibility, having no relevant cytotoxicity in tissues; hence, we decided to use it on wounded brain tissue [40]. However, we chose the use of antiseptic irrigation over antibacterial, even if evidence-based medicine lacks Class A evidence supporting the superiority of one over the other. Although in a large Cochrane Review conducted in 2017, which compared the use of antibacterial and non-antibacterial agents, the risk of SSI in general was lower in surgeries where antibacterial irrigation was employed, the studies presented low-class evidence [31]. In another trial conducted by Abiodun I. Okunola et al., antibacterial intraoperative irrigation with ceftriaxone did not alter the outcome [41].

Sodium hypochlorite, hypochlorous acid, and sodium chloride solutions, especially when used in intraoperative and postoperative settings, have demonstrated significant effectiveness in reducing the severity and incidence of infections in neurosurgical procedures. Future research should continue to refine these protocols, focusing on optimizing concentrations and application timings to maximize patient outcomes while minimizing adverse effects.

Prophylaxis for epilepsy in PBI is crucial due to the high risk of post-traumatic epilepsy (PTE), which occurs in approximately 30–50% of such cases [15,20]. The direct trauma to the cerebral cortex and subsequent scarring are primary contributors to this condition [13]. Prophylactic AEDs, such as sodium valproate, phenytoin, carbamazepine, or phenobarbital, are commonly used in the acute phase to minimize the incidence of PTE [15,20]. Studies emphasize that AEDs are most effective when administered within the first seven days post-injury, as extending their use beyond this period offers no added benefit in PTE prevention [13]. This early intervention aligns with the general management principles for PBIs, which aim to mitigate both immediate and long-term neurological complications [12].

Proper surgical management and infection control also play critical roles in reducing PTE risk. The removal of foreign objects, meticulous debridement, and dural repair are essential to minimize cortical irritation and scarring [24,25]. Prophylactic antibiotics are similarly critical, as infections like meningitis or abscesses exacerbate the risk of PTE [13,24]. Empirical regimens often include cephalosporins and metronidazole for seven days, as demonstrated in successful outcomes [13]. Early surgical intervention, combined with AEDs and antibiotics, ensures better seizure control and overall prognosis for PBI patients [12,15].

According to the current review, 52.6% of patients were neurologically intact at the last follow-up. Compared with PBIs caused by missiles or bullets, this can be considered a favorable outcome. In contrast, missile PBI rarely presents with an asymptomatic state, is characterized by high morbidity, and the mortality rate ranges between 23 to 93% according to a recent literature review [26]. A recent systematic review confirmed these findings, reporting a mortality rate between 66% and 83% in patients treated non-surgically and 13 and 33% in patients undergoing surgery [42].

In 18 patients from the current review, a neurological deficit remained following treatment of PBI. Impaired or complete loss of vision was the most common persistent neurological deficit and occurred mainly in patients with transorbital injuries. In this context, transmasal and transmental injuries were characterized by better outcomes. However, some patients developed complications, including CSF leaks, pneumocephalus, intracranial hemorrhages, and vascular injuries, which required additional interventions.

In the current review, CSF leak emerged as the most frequently reported complication, occurring in nine patients (22.0%). This rate is relatively high compared to the findings from three additional studies. Lan et al. reported CSF rhinorrhea or orbitorrhea in 4 out of 22 cases (18.2%) of non-missile anterior skull-base PBI, indicating a slightly lower incidence despite the complexity of the cases studied [12]. Yoneoka et al. documented a single case of CSF leakage following a transnasal anterior skull base fracture, managed successfully with a fat-on-fascia graft, reflecting the rarity of this complication in isolated cases [43]. Meanwhile, Khayat et al. identified CSF leakage in 6 out of 24 patients (25%) with crossbow-related head injuries, comparable to the current study [44].

Pneumocephalus, the presence of air within the cranial cavity, is a recognized complication of PBI, occurring in 7.3% of cases in our study. According to Hyung et al., pneumocephalus was noted in two cases: one involving extensive intracranial air following a sword injury and another from a drill injury. The former required surgical intervention with dural repair, while the latter was managed conservatively with antibiotics, both achieving good outcomes.

Our study reported an intracranial hemorrhage rate of 14.6%, which is lower compared to previous studies. Aljuboori et al., examining crossbow injuries, reported a 100% rate (3/3 cases) of intracranial hemorrhage, likely due to the high-energy mechanism of injury [10]. Lan et al., focusing on non-missile anterior skull-base injuries, found a rate of 18.2% (4/22 cases), closer to our findings but slightly higher [11,12]. Aydin reported a case of trauma from a fishing sinker, describing intracranial hemorrhages in the single reported patient, yielding a 100% rate. These variations reflect differences in injury mechanisms and patient populations across studies.

Vascular injuries occurred in 4.9% of patients in this series, emphasizing their significance in non-missile PBIs. Non-missile PBIs, though rare, present unique challenges due to potential neurovascular damage. Proper preoperative imaging, such as CT, CTA, or DSA, is critical for identifying vascular involvement and planning surgical approaches [15].

Considering the rarity of non-missile PBI, a small amount and low quality of available evidence regarding its management does not surprise. Thus, many knowledge gaps still exist, such as a lack of management guidelines or identified predictors of outcomes and complications, which can be explored by further studies. Moreover, the use of intraoperative irrigation with SHC/HCA-G is scarcely described in the literature. Further studies are needed to evaluate its efficacy compared to other substances used for antiseptic purposes.

## 5. Conclusions

Management of non-missile PBIs involving the anterior part of the skull base is complex, challenging, and often requires a multidisciplinary team including neurosurgeons, ENT surgeons, ophthalmologists, and maxillofacial surgeons. Comprehensive clinical examination, CT, and DSA or CTA imaging in suspected vascular injuries are essential for making a proper diagnosis. Moreover, the patients should be carefully observed for potential complications such as CSF leak, neuroinfection, or PTE. Therefore, antibacterial and antiseizure prophylaxis is highly recommended in this subclass of craniofacial injuries. In the treatment of patients with non-missile PBIs, following proper management may lead to favorable outcomes with minimal remaining symptoms or neurological deficits.

## Figures and Tables

**Table 1:** PubMed database search strategy.

No.	Search Query
#1	“Brain Injuries, Traumatic” [MeSH] OR “traumatic brain injur *” [tiab] OR “craniocerebral injur *” [tiab] OR “traumatic head injur *” [tiab] OR “neurotrauma *” [tiab] OR “cerebral injur *” [tiab] OR “brain injur *” [tiab]
#2	“Wounds, Stab” [MeSH] OR “nonmissile” [tiab] OR “non-missile” [tiab] OR “non missile” [tiab] OR “stab injur *” [tiab] OR “stabbing” [tiab] OR “foreign bod *” [tiab] OR “stab *” [tiab] OR “Head Injuries, Penetrating” [MeSH] OR “penetrat *” [tiab] OR “penetrating brain injur *” [tiab]
#3	“Cranial Fossa, Anterior” [MeSH] OR “anterior skull base” [tiab] OR “anterior skull-base” [tiab] OR “anterior cranial base” [tiab] OR “frontal base” [tiab] OR “frontal bone” [tiab] OR “ethmoid *” [tiab] OR “sphenoid *” [tiab] OR “anterior cranial fossa” [tiab] OR “skull * base” [tiab] OR “skull-base” [tiab]
#4	#1 AND #2 AND #3

**Table 2:** Characteristics of included studies.

Article No.	Authors	Year	Country	Case No.	Age	Gender	Mechanism	Injury Cause	Type of Injury	Type of Imaging	Type of Management	Other Specialists Assistance	Infection Prophylaxis	Outcome
	Current case	2024	Poland	1	20	M	suicide attempt	crossbow	transmental	CT	surgical	ENT surgeon	yes	olfactory dysfunction
1	Khayat	2024	Canada	2	31	M	suicide attempt	crossbow	transmental	CT and CTA	surgical	ENT surgeon	not reported	no deficit
2	Anwer	2024	India	3	32	M	accidental	sugarcane	transorbital	CT	surgical	ophtalmologist	yes	loss of vision
3	Hyung	2023	Korea	4	41	F	accidental	tree branch	transnasal	CT	surgical	-	yes	no deficit
				5	60	M	accidental	electric drill	transorbital	CT	non-operative	-	yes	no deficit
4	Al-Alousi	2022	Iraq	6	30	M	accidental	rifle rod	transnasal	CT and plain radiographs	surgical	maxillofacial surgeon and ENT surgeon	yes	no deficit
5	Widodo	2022	Indonesia	7	28	M	accidental	wooden stick	transnasal	CT and plain radiographs	surgical	ENT surgeon	yes	no deficit
6	Gupta	2022	India	8	24	F	accidental	pressure cooker nozzle	transorbital	CT and plain radiographs	surgical	-	not reported	loss of vision
7	Aljuboori	2022	United States	9	22	M	suicide attempt	crossbow	transmental	CT, CTA, and DSA	surgical	ENT surgeon	not reported	n/r
				10	67	M	suicide attempt	crossbow	transmental	CT, CTA, and DSA	surgical	ENT surgeon	not reported	n/r
				11	36	M	suicide attempt	crossbow	transmental	CT, MRI, CTA, and DSA	surgical	ENT surgeon	not reported	n/r
8	Yoneoka	2020	Japan	12	65	M	accidental	garden pole	transnasal	CT	non-operative	-	yes	no deficit
9	Prasetyo	2020	Indonesia	13	28	M	accidental	metal arrow	transorbital	CT, CTA, and plain radiographs	surgical	ophtalmologist	yes	loss of vision
10	Asadullah	2020	Indonesia	14	43	M	violence	wooden stick	transorbital	CT and CTA	surgical	ophtalmologist	yes	impaired vision
				15	18	M	accidental	fish bullet	transorbital	CT and CTA	surgical	ophtalmologist	yes	no deficit
				16	36	M	accidental	fish bullet	transorbital	CT	surgical	-	yes	no deficit
11	Aydin	2019	Turkey	17	37	M	accidental	fishing sinker	transorbital	CT	surgical	-	not reported	right hemiparesis
12	Lan	2018	China	18	51	M	accidental	wooden stick	transorbital	CT and MRI	surgical	-	yes	no deficit
				19	22	M	violence	metal rod	transorbital	CT and MRI	surgical	-	yes	no deficit
				20	30	M	accidental	wooden stick	transorbital	CT and MRI	surgical	-	yes	no deficit
				21	40	M	violence	wooden stick	transorbital	CT and MRI	surgical	-	yes	no deficit
				22	29	M	accidental	wooden stick	transorbital	CT and MRI	surgical	-	yes	right oculomotor palsy
				23	32	M	violence	knife	transorbital	CT and MRI	surgical	-	yes	right oculomotor palsy
				24	31	M	accidental	chopstick	transorbital	CT and MRI	surgical	-	yes	right abducens palsy
				25	44	M	accidental	wooden stake	transorbital	CT, MRI, and CTA	surgical	-	yes	no deficit
				26	43	M	accidental	bamboo stick	transorbital	CT, MRI	surgical	-	yes	no deficit
				27	32	M	violence	nail	transorbital	CT and MRI	non-operative	-	yes	no deficit
				28	42	M	accidental	wooden stick	transorbital	CT and MRI	surgical	-	yes	no deficit
				29	23	M	violence	pool cue	transorbital	CT and MRI	surgical	-	yes	loss of vision
				30	41	M	accidental	wooden stick	transnasal	CT and MRI	surgical	-	yes	loss of vision
				31	22	M	accidental	tree twig	transorbital	CT and MRI	surgical	-	yes	left oculomotor palsy
				32	51	M	accidental	tree twig	transorbital	CT and MRI	surgical	-	yes	no deficit
13	Zhang	2017	China	33	75	F	accidental	bamboo stick	transorbital	CT, MRI, and DSA	surgical	-	yes	no deficit
				34	42	M	accidental	electric drill	transorbital	CT	non-operative	-	yes	no deficit
				35	29	M	accidental	screw	transorbital	CT	surgical	-	yes	oculomotor nerve injury and impaired vision
				36	40	M	accidental	hot projective oil paint	transorbital	CT and MRI	surgical	-	yes	loss of vision
14	Li	2017	China	37	34	M	accidental	metal rod	transmental	CT and CTA	surgical	ENT surgeon	yes	loss of vision
				38	52	M	accidental	metal rod	transorbital	CT, CTA, and DSA	surgical	-	yes	coma
15	Chowdhury	2016	Bangladesh	39	45	M	violence	bamboo stick	transorbital	CT and plain radiographs	surgical	-	not reported	loss of vision
16	Tewari	2015	India	40	35	M	accidental	metal rod	transorbital	plain radiographs	surgical	-	yes	ptosis
17	Sweeney	2011	United States	41	31	M	suicide attempt	knife	transmental	CT and CTA	surgical	ENT surgeon	not reported	no deficit

**Table 3:** Risk of bias assessment. Evaluated domains according to the JBI’s critical appraisal checklist tool were as follows: 1—Were the patient’s demographic characteristics clearly described?; 2—Was the patient’s history clearly described and presented as a timeline?; 3—Was the current clinical condition of the patient on presentation clearly described?; 4—Were diagnostic tests or assessment methods and the results clearly described?; 5—Was the intervention(s) or treatment procedure(s) clearly described?; 6—Was the post-intervention clinical condition clearly described?; 7—Were adverse events (harms) or unanticipated events identified and described?; 8—Does the case report provide takeaway lessons?

No.	Author	Year	1	2	3	4	5	6	7	8
1	Khayat	2024	Yes	Yes	Yes	Yes	Yes	Unclear	Yes	Yes
2	Anwer	2024	Yes	Yes	Yes	Yes	Yes	Yes	Yes	Unclear
3	Hyung	2023	Yes	No	Yes	Yes	No	Unclear	Yes	Yes
4	Al-Alousi	2022	Yes	Yes	Yes	Yes	No	Yes	Yes	No
5	Widodo	2022	Yes	Yes	Yes	Yes	Yes	Yes	Yes	Yes
6	Gupta	2022	Yes	Yes	Yes	Yes	Yes	Yes	Yes	Yes
7	Aljuboori	2022	Yes	Yes	Yes	Yes	Yes	No	Yes	Yes
8	Yoneoka	2020	Yes	Yes	Yes	Yes	Yes	Yes	Yes	Yes
9	Prasetyo	2020	Yes	Yes	Yes	Yes	Yes	Yes	Yes	Yes
10	Asadullah	2020	Yes	Yes	Yes	Yes	Yes	Yes	Yes	Yes
11	Aydin	2019	Yes	No	Yes	Yes	Unclear	Yes	Yes	No
12	Lan	2018	Yes	Unclear	Yes	Yes	Yes	Yes	Yes	Unclear
13	Zhang	2017	Yes	Yes	Yes	Yes	Yes	Yes	Yes	Yes
14	Li	2017	Yes	No	Yes	Yes	Yes	Yes	Yes	Yes
15	Chowdhury	2016	Yes	Yes	Yes	Yes	Yes	Yes	Yes	No
16	Tewari	2015	Yes	Yes	Yes	Yes	Yes	Yes	Yes	Unclear
17	Sweeney	2011	Yes	No	Yes	Yes	Yes	Yes	Yes	Yes

**Table 4:** Baseline characteristics of adult patients with non-missile anterior skull base PBI; Abbreviations: GCS—Glasgow Coma Scale.

Variable	n (%)			
	Total, n = 41	Transorbital, n = 29	Transmental, n = 7	Transnasal, n = 5
**Age (years)**	37.4 ± 13.1	37.5 ± 12.5	34.4 ± 15.5	41.0 ± 14.7
**Gender**				
Male	38 (92.7%)	27 (93.1%)	7 (100.0%)	4 (80.0%)
Female	3 (7.3%)	2 (6.9%)	0 (0.0%)	1 (20.0%)
**Mechanism of injury**				
Accident	28 (68.3%)	22 (75.9%)	1 (14.3%)	5 (100.0%)
Violence	7 (17.1%)	7 (24.1%)	0 (0.0%)	0 (0.0%)
Suicide attempt	6 (14.6%)	0 (0.0%)	6 (85.7%)	0 (0.0%)
**Neurological function on admission**				
Intact	30 (73.2%)	20 (69.0%)	7 (85.7%)	4 (80.0%)
Impaired	11 (26.8%)	9 (31.0%)	1 (14.3%)	1 (20.0%)
**GCS on admission (reported data)**	**n = 37**	**n = 26**	**n = 6**	**n = 5**
15	20 (54.1%)	10 (38.5%)	6 (100.0%)	4 (80.0%)
Below 15	17 (45.9%)	16 (61.5%)	0 (0.0%)	1 (20.0%)

**Table 5:** Imaging modalities, treatment methods, and outcomes in non-missile anterior skull base PBI. Abbreviations: CT—computed tomography, MRI—magnetic resonance imaging, DSA—digital subtraction angiography, and CTA—computed tomography angiography.

Variable	n (%)			
	Total, n = 41	Transorbital, n = 29	Transmental, n = 7	Transnasal, n = 5
**Imaging**				
Plain radiographs	6 (14.6%)	4 (13.8%)	0 (0.0%)	2 (40.0%)
CT	40 (97.6%)	28 (96.6%)	7 (100.0%)	5 (100.0%)
MRI	18 (43.9%)	16 (55.2%)	1 (14.3%)	1 (20.0%)
DSA	5 (12.2%)	2 (6.9%)	3 (42.8%)	0 (0.0%)
CTA	11 (26.8%)	5 (17.2%)	6 (85.7%)	0 (0.0%)
**Type of treatment**				
Operative	37 (90.2%)	26 (89.7%)	7 (100.0%)	4 (80.0%)
Non-operative	4 (9.8%)	3 (10.3%)	0 (0.0%)	1 (20.0%)
**Antimicrobial prophylaxis**	33 (80.5%)	27 (93.1%)	1 (14.3%)	5 (100.0%)
**Operative field irrigation with antibiotics/antiseptics**	2 (4.9%)	1 (3.4%)	1 (14.3%)	0 (0.0%)
**Antiseizure prophylaxis (reported data)**	**n = 7**	**n = 5**	**n = 1**	**n = 1**
Phenytoin	2 (28.6%)	2 (40.0%)	0 (0.0%)	0 (0.0%)
Sodium valproate	2 (28.6%)	1 (20.0%)	1 (100.0%)	0 (0.0%)
Not specified	3 (42.8%)	2 (40.0%)	0 (0.0%)	1 (100.0%)
**Outcome at last follow-up (reported data)**	**n = 38**	**n = 29**	**n = 5**	**n = 4**
Neurologically intact	20 (52.6%)	14 (48.3%)	4 (80.0%)	2 (50.0%)
Persisted deficit	18 (47.4%)	15 (51.7%)	1 (20.0%)	2 (50.0%)

## Data Availability

Not applicable.

## Supplementary Materials

The following supporting information can be downloaded at: https://www.mdpi.com/article/10.3390/jcm14165731/s1, File S1: The PRISMA checklist [45].
